# Anisole–Water and Anisole–Ammonia Complexes in Ground and Excited (S_1_) States: A Multiconfigurational Symmetry-Adapted Perturbation Theory (SAPT) Study

**DOI:** 10.1021/acs.jpca.4c04928

**Published:** 2024-10-01

**Authors:** Agnieszka Krzemińska, Malgorzata Biczysko, Katarzyna Pernal, Michał Hapka

**Affiliations:** † Institute of Physics, Lodz University of Technology, ul. Wolczanska 217/221, 93-005 Lodz, Poland; ‡ Faculty of Chemistry, University of Wroclaw, F. Joliot-Curie 14, 50-383 Wroclaw, Poland; ¶ Faculty of Chemistry, University of Warsaw, ul. L. Pasteura 1, 02-093 Warsaw, Poland

## Abstract

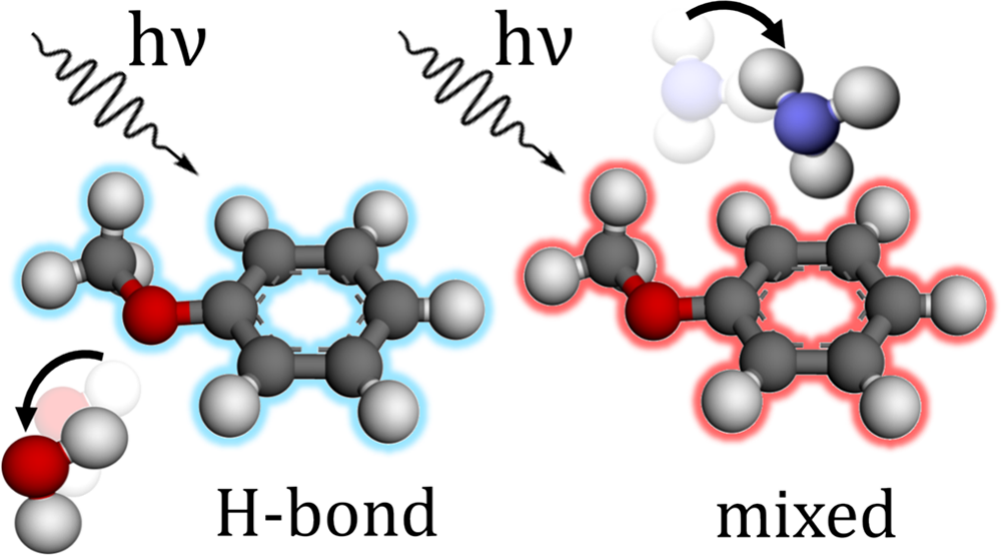

Binary complexes of anisole have long been considered paradigm systems for studying microsolvation in both the ground and electronically excited states. We report a symmetry-adapted perturbation theory (SAPT) analysis of intermolecular interactions in anisole–water and anisole–ammonia complexes within the framework of the multireference SAPT(CAS) method. Upon the S_1_ ← S_0_ electronic transition, the hydrogen bond in the anisole–water dimer is weakened, which SAPT(CAS) shows to be determined by changes in the electrostatic energy. As a result, the water complex becomes less stable in the relaxed S_1_ state despite decreased Pauli repulsion. Stronger binding of the anisole–ammonia complex following electronic excitation is more nuanced and results from counteracting shifts in the repulsive (exchange) and attractive (electrostatic, induction and dispersion) forces. In particular, we show that the formation of additional binding N–H···π contacts in the relaxed S_1_ geometry is possible due to reduced Pauli repulsion in the excited state. The SAPT(CAS) interaction energies have been validated against the coupled cluster (CC) results and experimentally determined shifts of the S_1_ ← S_0_ anisole band. While for the hydrogen-bonded anisole–water dimer SAPT(CAS) and CC shifts are in excellent agreement, for ammonia SAPT(CAS) is only qualitatively correct.

## 1 Introduction

Characterizing noncovalent interactions in isolated molecular clusters is essential for understanding a wide range of phenomena, from molecular reactions in the cold regime^1,2^ to supramolecular chemistry and molecular self-assembly.^3,4^ While enormous progress has been made in developing quantum algorithms for accurate prediction and analysis of noncovalent interactions, most works have so far focused on ground-state properties. Yet, the design of novel materials, such as high-performance organic semiconductors, often requires accessing excited states.^5,6^ In some cases, the knowledge of the ground-state interactions may already be sufficient for fine-tuning the desired properties. For instance, minimizing the Pauli repulsion between the frontier orbitals closes the singlet–triplet gap in thermally activated delayed fluorescence emitters.^7,8^ In a similar vein, combining phosphor and emission-inducing agents via halogen bonding may induce room-temperature phosphorescence via spin–orbit coupling.^9^ However, studies that give a direct insight into the role of noncovalent interactions in excited-state complexes are still scarce.^10^

The theoretical description of excited-state interactions is more complex and computationally demanding compared to ground states. This is particularly difficult because states of different nature (e.g., valence, Rydberg, charge transfer) are often close in energy.^11^ The supermolecular approach to the interaction energy is no longer straightforward in regions with a high density of states. For instance, basis sets with diffuse functions, a prerequisite for describing dispersion interactions, capture low-lying Rydberg states which may interact with the target valence states. Moreover, the counterpoise (CP) correction scheme^12^ that corrects for the basis set superposition error (BSSE) fails in the case of delocalized excitons for which charge transfer effects are pronounced.^13^ Another problem is that standard dispersion corrections routinely applied in DFT calculations are no longer suitable for treating excited states^14,15^ as they rely on parameters tuned on ground-state properties.

A particular challenge is interpreting the nature of intermolecular forces in excited-state complexes. Several energy decomposition analysis (EDA) approaches have already been developed to address this issue. Ge and Head-Gordon^16,17^ proposed absolutely localized molecular orbital (ALMO) EDA scheme based on configuration interaction singles (CIS) or time-dependent density functional theory (TD-DFT) treatment of excited states. The ALMO-EDA scheme was first developed for systems with localized excitons (exciplexes),^16^ and soon after extended to excimers.^17^ Su and co-workers^18^ introduced a semiempirical EDA formulated in the density functional based tight binding (DFTB) framework. Combined with time-dependent DFTB,^19^ the method was applied to several model, medium-size exciplexes featuring hydrogen bonds and OH···π interactions.^18^ Recently, Su et al.^20^ extended the generalized Kohn–Sham EDA (GKS-EDA)^21^ based on TD-DFT to excited states. A different EDA approach rooted in multistate DFT^22^ was presented in refs (23 and 24). By introducing intermediate diabatic states that could be variationally optimized, the authors separated excited-state binding energies into exciton coupling, superexchange (charge transfer), and delocalization effects.

Symmetry-adapted perturbation theory (SAPT)^25,26^ provides a viable and successful alternative to variational EDAs. Unlike EDAs, SAPT is capable of providing both a rigorous, perturbation-theory based, analysis of molecular interactions and their highly accurate values.^27^ Recently, we have developed a multiconfigurational (MC) variant of symmetry-adapted perturbation theory applicable to both ground- and excited-state interactions with localized excitons.^28−30^ The method, referred to as SAPT(MC), employs perturbation expansion through second order in the intermolecular interaction operator and can be combined with any approach that provides one- and two-electron reduced density matrices of monomers. This is possible by computing monomer response properties within the extended random phase approximation^31,32^ and limiting all exchange terms to the single-overlap (*S*^2^) approximation. Combined with the complete active space (CAS) wave function, SAPT(MC) has proven useful in analyzing noncovalent interactions in model complexes involving localized excitons.^33,34^

Aromatic chromophores belong to systems of choice for probing noncovalent interactions in both ground and excited states, since they can be spectroscopically detected using fluorescence or resonant ionization methods.^10,35−37^ Anisole is a prototypical polar chromophore, capable of forming hydrogen bonds, with both the oxygen atom of methoxy group (−OCH_3_) and aromatic π-electrons acting as potential acceptor sites. Additionally, anisole may participate in π–π stacking and OH···π interactions due to the presence of an aromatic ring. The interplay of intermolecular forces in anisole-containing clusters has been widely studied with a variety of experimental techniques including molecular beams,^38,39^ velocity map imaging^40^ and superfluid helium nanodroplets.^41^ Combined experimental and theoretical studies identified anisole complexes spanning practically the entire spectrum of noncovalent binding patterns. From van der Waals systems bound by dispersion, such as anisole–argon clusters^39^ and the anisole dimer,^42−46^ to hydrogen-bonded complexes, with anisole–water^47−49^ providing a paradigm example. Numerous complexes of mixed character, where both electrostatics and dispersion contribute to bonding, were also described, including binding of ammonia,^50−53^ carbon dioxide^54,55^ and (CO_2_)_2_,^39^ among others.^56,57^ Different docking scenarios lead to closely lying minima, such as competition between OH···O and OH···π hydrogen bonds in the anisole–methanol dimer.^41,58^ In many cases, it was possible to gain insight into the nature of excited-state interactions by measuring the band shifts of the first bright singlet electronic transition in the cluster with respect to that of the bare chromophore, where blue and red shifts correspond to weaker and stronger binding in the excited state, respectively.

In this work, we focus on two prototype binary complexes of anisole: anisole–water and anisole–ammonia. The water complex raised interest as the first system in molecular beam experiments where water was observed acting as an acid, i.e., as a proton donor in a hydrogen bond.^47^ The structure of the dimer both in ground S_0_ and S_1_ excited states was established throughout the 2000s based on rotationally resolved spectra, including fluorescence^47−49^ and microwave^59^ studies supported by theoretical calculations. The hydrogen bond which stabilizes the complex is formed between the water hydrogen atom and the anisole oxygen, with O–H···O atoms lying in plane with the aromatic ring. The complex exhibits a significant structural change upon ^1^H/^2^H isotopic substitution^37,49,59^ attributed both to dynamic effects and the O–H/D bond length variation, affecting H_2_–O···H–C secondary interactions. In contrast to water, the anisole–ammonia dimer is nonplanar. As verified by rotationally resolved electronic^50,51^ and microwave^52^ spectra, the complex in the ground state is stabilized by three contacts of the N–H···π, N–H···O and C–H···N types. The computational study of Barone and co-workers^60^ showed that both the structure and binding energies of anisole–ammonia are highly sensitive to the level of theory. As indicated by a SAPT analysis,^60^ the large spread of results obtained with various density functional approximations can be attributed to the pronounced dispersion interactions in this complex. The discrepancy between coupled-cluster (CC) and experimental binding energies persists to this day—a recent two-color appearance potential (2CAP) measurement of Reid et al.^53^ reported binding energy of 10.2(7) kJ/mol, which is rather far from 7.10 kJ/mol obtained in subsequent basis-set extrapolated CCSD(T) calculations of ref (61).

Measurements of the origin S_1_ ← S_0_ band shift relative to the isolated anisole revealed different behavior of water and ammonia 1:1 complexes upon electronic excitations. For water, a blue shift^47^ of 119 cm^–1^ was observed corresponding to a decrease in binding, while for ammonia a red shift^50^ of −199 cm^–1^ indicates stronger noncovalent interactions in the S_1_ state. Changes in binding patterns and geometry relaxation in the S_1_ state are explained by invoking the orbital picture: exciton located on anisole corresponds to the transfer of the electron density from the oxygen atom on the methoxy-group to the π system.^49,51^ In water, this weakens the hydrogen bond and makes the secondary interaction between the water oxygen atom and methyl group on anisole more pronounced, as related to variations in interatomic distances and natural bond orbital analysis.^49^ In ammonia, the electron density rearrangement on anisole results in lengthening of the N–H···O distance and formation of two close N–H···π contacts compared to a single one in the ground state.^51^

Our work presents a SAPT analysis of anisole–water and anisole–ammonia complexes in their ground state and first singlet excited states (both vertical and adiabatic), with excitons localized on the anisole molecule. This is possible using the SAPT(MC) approach, which in this work is applied with CASSCF monomer wave functions and is specifically referred to as SAPT(CAS). A rigorous examination of SAPT interaction energy components elucidates changes in binding patterns triggered by vertical excitation and the subsequent geometry relaxation. The SAPT picture viewing the interaction through fundamental electrostatic, induction, dispersion, and Pauli repulsion (exchange) forces complements previous, orbital-based interpretations, providing further insight. For the sake of comparison, we refer to the CCSD(T) and EOM-CCSD results, as well as the experimental shifts. Additionally, the numerical study is enriched by visualizing changes in the local dispersion energy descriptor.^34^

## 2 Computational Details

**Figure 1:**
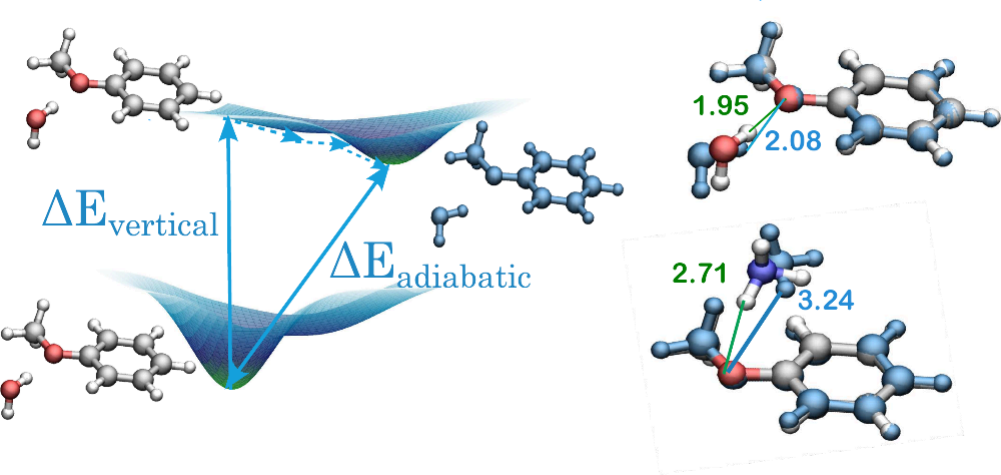
Schematic representation of vertical and adiabatic electronic excitations depicted on the example of anisole–H_2_O complex with differences between ground and excited state (blue color coded) geometries for anisole–H_2_O and anisole–NH_3_ complexes. Distances in Å.

The ground (S_0_) state geometries of the anisole–water and anisole–ammonia complexes were taken from refs (49 and 51), respectively (B3LYP/6-311+G(d,p) and CP-corrected MP2/6-311+G(d,p), respectively). For both complexes, geometries of the first singlet excited state (S_1_) were taken from ref (49) (optimized at the TD-B3LYP/6-311+G(d,p) level of theory). Following the work of Barone et al.,^60^ we chose structures that most accurately reproduce the experimental rotational constants for both systems. Upon excitation, the anisole ring bends slightly, the interacting partner molecule rotates, and the distance between the anisole oxygen atom and the closest hydrogen atom of the small molecule elongates (see Figure 1).

SAPT(CAS)^30^ calculations based on CASSCF treatment of the monomers were performed in the GammCor(62) program. The necessary integrals and one- and two-electron reduced density matrices were obtained from the locally modified Molpro^63^ package. SAPT2+(3) calculations were performed in the Psi4^64^ code. Excitation energies EOM-CCSD calculations as well as supermolecular CASSCF, MP2 and CCSD(T) calculations were performed in Molpro. The aug-cc-pVXZ (X = T, Q) Dunning^65,66^ basis sets were used throughout. Supermolecular interaction energies were obtained employing the Boys–Bernardi counterpoise correction.^12^

State-specific CASSCF was used for both ground- and excited state complexes. (For excited states, we performed state-averaged two-state CASSCF calculations with weights equal to 0 and 1 for ground and excited states, respectively.) In SAPT(CAS) calculations, we chose CAS(8,8) active space with the 1s core orbital inactive for both water and ammonia. For anisole, we selected CAS(12,12) active space including three π and three π* orbitals on the benzene ring, π_OC_ and π_OC_^*^ orbitals, two σ_CO_ together with their antibonding σ_CO_^*^ partners, see Figure S1 in the Supporting Information. In Table S1 in the Supporting Information, we report total CASSCF ground- and excited-state energies for both complexes.

Supermolecular CASSCF calculations were performed in a smaller CAS(6,6) active space on anisole (limited to π electrons), with the water or ammonia described using a Hartree–Fock reference. As a guess for the dimer CASSCF wave function, we employed merged orbitals from monomer CAS(6,6)SCF and RHF calculations (see also Tables S2 and S3 in Supporting Information for SAPT(CAS) results in the smaller active space).

For visualization purposes, the dispersion energy descriptor^34^ was computed using CASSCF orbitals localized according to the Pipek–Mezey^67^ scheme. The descriptor, inspired by refs (68−70), is a spatially local dispersion energy density function, , which integrates to the dispersion energy, *E*_disp_^(2)^ = ∫*Q*^AB^(**r**) d**r**. The monomer *Q*^*X*^ functions are obtained by summing densities of occupied natural orbitals weighted by their contribution to the dispersion energy. For details, see ref (34).

The choice of the active space in SAPT(CAS) and supermolecular CASSCF calculations requires a comment. Selection of the CAS(12,12) active space for anisole in SAPT(CAS) is motivated by the observation that the absence of π_OC_ and σ_CO_ orbitals (with their antibonding partners, cf. Figure S1 in the Supporting Information) leads to a poor, Hartree–Fock like, description of first-order energy contributions in the anisole–water dimer. Single-reference SAPT calculations accounting for intramonomer correlation effects [SAPT(DFT) and SAPT2+(3)] reveal that, in the ground state, the exchange energy outweighs electrostatics in magnitude, while the opposite is true at the SAPT0 level of theory. SAPT(CAS) results for the anisole–water complex based on the CAS(6,6) reference match SAPT0 (see Table S2 in the Supporting Information). Extending the active space to CAS(12,12) improves first-order SAPT(CAS) energies. In the case of supermolecular CASSCF calculations, in the discussion we refer to the CAS(6,6)-based results as the example of an approach with practically neglects intramonomer correlation effects in the interaction energy. CAS(6,6) includes only π orbitals on the carbon ring and it is a minimal active space necessary to describe the first valence excited state of anisole.

The interaction energy in SAPT(CAS)^30^ is calculated as a sum of contributions through second order in the interaction operator
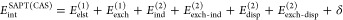
1where terms on the right-hand side are first-order electrostatic and exchange energies, second-order induction and dispersion energies, and their exchange counterparts. All exchange components are computed in the *S*^2^ approximation. The δ term approximates induction effects beyond the second-order in the interaction operator. For ground-state complexes, these were approximated at the Hartree–Fock level of theory and denoted as δ_HF_:^71,72^
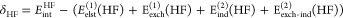
2where each SAPT component was computed with Hartree–Fock wave functions, while *E*_int_^HF^ corresponds to the supermolecular Hartree–Fock interaction energy. It is reasonable to assume that the ratios of the induction energy in the valence excited state to that in the ground state are equal in all orders. Therefore, higher-than-second-order induction energy in the considered excited states was approximated via a δ_CAS_ correction:^33^
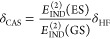
3where labels GS and ES correspond to dimers in the ground and excited states, respectively, and *E*_IND_^(2)^ = *E*_ind_^(2)^ + *E*_exch-ind_^(2)^.

The SAPT(CAS) results in the complete basis set (CBS) limit were obtained with a composite approach. First-order electrostatic and exchange energies, as well as second-order induction effects were calculated in the aug-cc-pVQZ basis set. Both dispersion and exchange-dispersion energies were extrapolated to the CBS limit from aug-cc-pVTZ (AVTZ) to aug-cc-pVQZ (AVQZ) basis sets using the two-point scheme of Halkier et al.^73^ Since second-order exchange terms in SAPT(CAS) require solving the full ERPA eigenproblem^29^ that entails *N*^6^ computational cost with the system size *N*, their values in the quadrupole-zeta basis were approximated by scaling of the uncoupled^29^ counterparts:
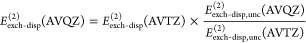
4and analogous formula for the exchange-induction energy.

For ground states, the SAPT(CAS) results will be compared to those of the SAPT2+(3) method.^74^ The latter includes contributions from intramonomer correlation through a double-perturbation expansion and accounts for the leading third-order term in the dispersion energy
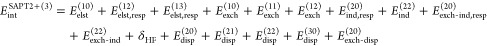
5where the second superscript refers to the order in the intramonomer perturbation operator, and “resp” stands for relaxation (response) of the monomer orbitals in the electric field of the partner. As is customary, in the following we will refer to the sums of terms in the intramonomer perturbation by omitting the second index, e.g., *E*_elst_^(1)^ will denote the sum of *E*_elst_^(10)^, *E*_elst,*resp*_^(12)^, and *E*_elst,*resp*_^(13)^.

As the reference for ground-state interaction energies, we report CCSD(T) results extrapolated to the CBS limit according to
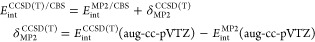
6where the CBS extrapolation of the MP2 interaction energy was performed according to CBS(T,Q) scheme of ref (73), the same as used for the dispersion energy extrapolation. Reference excited-state interaction energies were calculated by supplementing ground-state *E*_int_^CCSD(T)/CBS^ energies with a Δ_EOM-CCSD_ shift computed in the aug-cc-pVTZ basis set according to
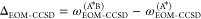
7where ω_EOM-CCSD_^(X)^ denotes the EOM-CCSD excitation energy of a compound *X*, i.e. either the anisole molecule (A*) or its dimer (A*B). To account for BSSE^12^ in supermolecular interaction energies, all calculations (including the EOM-CCSD excitation energy) for monomers were carried out in the dimer basis set.

## 3 Results

In Table 1, we present SAPT interaction energy decomposition for ground-state anisole–water and anisole–ammonia dimers. Using the dispersion/electrostatics ratio,^75^ equal to 0.6 and 1.2 for water and ammonia complexes, respectively, the former can be classified as hydrogen-bonded, while the interaction between anisole and ammonia is of a mixed character. Relative deviations between SAPT(CAS) and SAPT2+(3) energy components do not exceed 10%, and are larger for the water complex (note that the exchange-dispersion energy is more accurate in SAPT(CAS), since SAPT2+(3) includes only the uncoupled value of this term). The difference between the two SAPT approaches gives an estimate of the magnitude of intramonomer correlation effects that are included in SAPT2+(3), but practically neglected at the SAPT(CAS) level of theory. Compared to SAPT2+(3), the SAPT(CAS) approach consequently underestimates the magnitude of the leading attractive (dispersion, induction) and repulsive (first-order exchange) forces. Total interaction energies from both SAPT schemes remain in close agreement—for the water complex the difference amounts to less than 0.04 m*E*_h_, in the case of ammonia the interaction predicted by SAPT2+(3) is stronger by ca. 0.3 m*E*_*h*_. SAPT(CAS) overbinds water and ammonia dimers only by 6% and 4%, respectively, compared to supermolecular CCSD(T)/CBS results. As expected, the dispersion interaction, in both dimers exceeding 6 m*E*_h_ in magnitude, gives a substantial contribution to the binding. This is also reflected in a severe underestimation of supermolecular CASSCF interaction energies shown in Table 1 (recall that CASSCF lacks dispersion if the active orbitals are localized only on one of the monomers^76^).

**Table 1:** Comparison of SAPT, CASSCF and CCSD(T) Interaction Energies for Ground-State Anisole–Water and Anisole–Ammonia Complexes and SAPT Energy Components^a^

	anisole–H_2_O		anisole–NH_3_	
ground state	SAPT(CAS)	SAPT2+(3)	SAPT(CAS)	SAPT2+(3)
*E*_elst_^(1)^	–11.51	–11.63	–5.541	–5.523
*E*_exch_^(1)^	11.53	12.76	6.011	6.215
*E*_ind_^(2)^	–5.021	–5.587	–2.138	–2.191
*E*_exch-ind_^(2)^	2.950	3.060	1.534	1.547
*E*_disp_^(2)^	–6.134	–6.727	–6.510	–6.908
*E*_exch-disp_^(2)^	1.000	0.905	0.859	0.759
δ_HF_	–1.445	–1.445	–0.441	–0.441
*E*_int_^SAPT^	–8.629	–8.671	–6.226	–6.540
*E*_int_^CASSCF^	–4.858		–0.862	
*E*_int_^CCSD(T)^	–8.152		–6.082	

a All results are extrapolated to the CBS(T,Q) limit. For comparison, all exchange terms in SAPT2+(3) are given in the *S*^2^ approximation and the interaction energy does not include the *E*_disp_^(30)^ term. Energy unit is 1 m*E*_h_.

Although the lowest electronic excitation of the considered anisole complexes is primarily of the π–π* character, there is also a significant contribution from the n → π* transition. As a consequence of the latter, upon vertical excitation, the hydrogen bond in the anisole–water dimer is weakened, which translates into a 0.41 m*E*_*h*_ decrease in the electrostatic attraction (see Table 2). This effect dominates, as the respective changes in the exchange and induction components are 5–8 times smaller in magnitude, while the dispersion energy stays virtually the same. Geometry relaxation in the excited state leads to sizable changes of all SAPT(CAS) interaction energy components, cf. Tables 2 and 3. The spatial rearrangement of the dimer leading to further H-bond weakening (see Figure 1) significantly reduces Pauli repulsion—the first-order exchange energy drops by −3.29 m*E*_h_ compared to the excited-state dimer in the ground state geometry (confront the “adia–vert” column in Table 2). Yet, this effect is overshadowed by a simultaneous drop in all attractive interaction energy components, the most sizable being the decrease in the electrostatic energy, which changes by +1.90 m*E*_h_. Overall, relaxation of the geometry leads to a 0.20 m*E*_h_ destabilization of the excited-state complex: the excited-state SAPT(CAS) interaction energies in the vertical and adiabatic geometries are equal to −8.22 m*E*_h_ and −8.02 m*E*_h_, respectively, compared to −8.63 m*E*_h_ ground-state interaction energy. This matches supermolecular CASSCF results which predict a decrease in binding by 0.3 m*E*_h_ when the geometry in the S_1_ state is relaxed. In contrast, at the CC level of theory the interaction energy is strengthened by 0.4 m*E*_h_ upon geometry relaxation, as the excited-state interaction energies amount to −7.02 m*E*_h_ and −7.44 m*E*_h_ in the vertical and adiabatic geometries, respectively (the ground-state reference is −8.15 m*E*_h_). We attribute the difference between SAPT(CAS), CASSCF and CC calculations to the lack of electron correlation effects in CASSCF monomer wave functions.

**Table 2:** Differences of SAPT(CAS) Interaction Energy Components between Excited (ES) and Ground States (GS), Δ*E*_*x*_ = *E*_*x*_(ES) – *E*_*x*_(GS), for Vertical (vert) and Adiabatic (adia) Excitations^a^

	anisole–H_2_O			anisole–NH_3_		
	vert	adia	adia–vert	vert	adia	.adia–vert
Δ*E*_elst_^(1)^	0.408	2.307	1.899	0.172	–2.593	–2.765
Δ*E*_exch_^(1)^	–0.081	–3.375	–3.294	–0.110	4.806	4.916
Δ*E*_IND_^(2)^	0.051	0.602	0.551	0.011	–0.528	–0.539
Δ*E*_DISP_^(2)^	0.006	0.505	0.499	0.040	–1.202	–1.242
δ_HF/CAS_	0.036	0.584	0.548	0.008	–0.592	–0.600
Δ*E*_int_^SAPT^	0.420	0.623	0.203	0.121	–0.110	–0.231
Δ*E*_int_^CASSCF^	0.092	0.388	0.296	0.090	1.301	1.211
Δ_EOM-CCSD_	1.131	0.711	–0.420	–0.199	–1.224	–1.025

a *E*_IND_^(2)^ = *E*_ind_^(2)^ + *E*_exch-ind_^(2)^ and *E*_DISP_^(2)^ = *E*_disp_^(2)^ + *E*_exch-disp_^(2)^. Energy unit is 1 m*E*_h_.

**Table 3:** Comparison of SAPT, CASSCF, and CC Interaction Energies for Excited-State Anisole–Water and Anisole–Ammonia Complexes^a^

	anisole–H_2_O		anisole–NH_3_	
	vert	adia	vert	adia
*E*_elst_^(1)^	–11.10	–9.204	–5.369	–8.134
*E*_exch_^(1)^	11.45	8.157	5.901	10.82
*E*_ind_^(2)^	–4.961	–3.355	–2.118	–4.688
*E*_exch-ind_^(2)^	2.941	1.886	1.525	3.556
δ^CAS^	–1.419	–0.871	–0.433	–1.033
*E*_disp_^(2)^	–6.119	–5.392	–6.455	–8.313
*E*_exch-disp_^(2)^	0.991	0.763	0.844	1.460
*E*_int_^SAPT^	–8.219	–8.016	–6.105	–6.336
*E*_int_^CASSCF^	–4.767	–4.470	–0.773	0.433
*E*_int_^CCSD(T)^ + Δ_EOM-CCSD_	–7.021	–7.441	–5.883	–7.306

a Columns denoted as “vert” and “adia” refer to S_0_ and S_1_ geometries, respectively. SAPT energy components are also presented. Results extrapolated to the CBS(T,Q) limit. Energy unit is 1 m*E*_h_.

The anisole–ammonia dimer is distinctly different from the water complex in several aspects. First, the change in the interaction energy upon vertical excitation results from an interplay between all energy components, in contrast to the water binding where electrostatics clearly dominates. As shown in Table 2, in the case of ammonia the weakened electrostatics (0.17 m*E*_h_ difference with respect to the ground state) is offset by a comparable reduction in the exchange repulsion (−0.11 m*E*_h_). The decrease in the dispersion attraction is larger than in the anisole–water dimer and amounts to 0.04 m*E*_h_. A sum of the electrostatics, exchange, and dispersion effects is a major contributor to the overall 0.12 m*E*_h_ destabilization of the vertically excited-state complex with ammonia.

The second striking difference with respect to the water case is that geometry relaxation in the S_1_ exited state leads to an increase in binding of ammonia. In the SAPT(CAS) picture, the relaxed geometry favors the region of PES with a stronger overlap between the monomers: the magnitude of all energy components is notably larger compared to the ground state. While the repulsive exchange component increases by 4.81 m*E*_h_, the attractive components prevail, leading to the interaction energy increased by 0.11 m*E*_h_ compared to the ground state. Note that the opposite is true for water, where the relaxed geometry corresponds to both weaker attraction and the reduced Pauli repulsion, the latter effect being more important. The different behavior of S_1_ exited states upon geometry relaxation is reflected in the opposite signs of the Δ*E* terms for water and ammonia complexes in Table 2 (see the “adia” column).

We have shown that the increased interaction energy in the S_1_ state of ammonia relative to the ground state can be attributed both to geometry relaxation and electronic excitation. The distinct nature of the excited state can be also illustrated by considering vertical de-excitation, i.e., S_1_ → S_0_ electronic transition at the adiabatic geometry. The most significant change in SAPT(CAS) energy components for this process is the increase of Pauli repulsion which amounts to 1.0 m*E*_h_, first-order exchange alone contributing 0.6 m*E*_h_ (see Table 3 and Table S5 in the Supporting Information). This shows that electronically excited anisole features reduced exchange, compared to the ground state, which can accommodate two N–H···π contacts compared to a single N–H···π interaction in the S_0_ ground state.

In SAPT(CAS), the stabilization of the relaxed S_1_ state in the anisole–ammonia complex amounts to −0.11 m*E*_h_. Notably, this value deviates significantly from the EOM-CCSD result of −1.14 m*E*_h_ reported in Table 2. We attribute this discrepancy to the lack of intramonomer correlation effects in the CASSCF monomer wave functions. This conjecture is supported by CASSCF-based calculations with a smaller, CAS(6,6), active space on anisole. SAPT(CAS) calculations with a CAS(6,6) active space give a qualitatively wrong answer by predicting a stronger binding in the ground S_0_ state (see Table S3 in the Supporting Information). The same ordering is obtained with supermolecular CAS(6,6)SCF calculations, (see Table 3). Even after supplementing CAS(6,6)SCF with the dispersion energy,^76^ the S_0_ state remains ca. 0.1 m*E*_h_ more strongly bound compared to S_1_. These results confirm that the size of the active space used for monomer descriptions, which is directly related to the amount of the captured intramonomer electron correlation energy, plays a major role in predicting direction of changes of the binding energy upon excitation in the anisole–ammonia complex.

**Figure 2:**
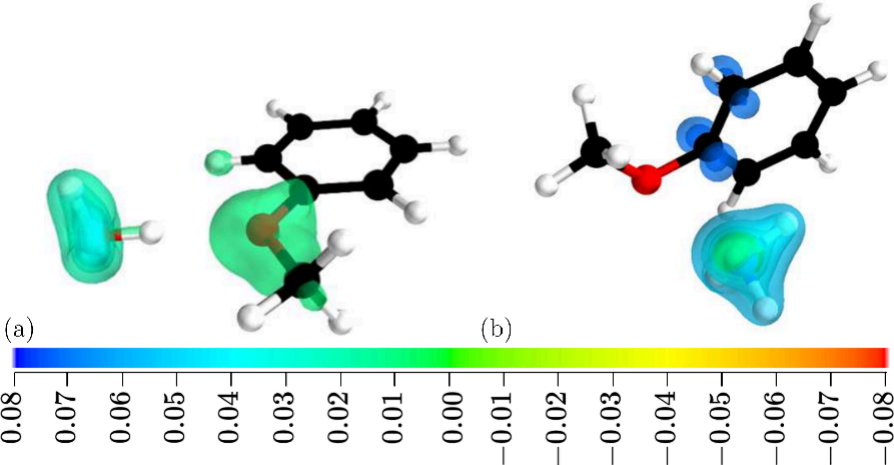
Difference of the dispersion energy density Δ*Q*^AB^(**r**) between vertical excited-state and ground-state Δ*Q*^AB^(**r**) = *Q*^A^*^B^(**r**) – *Q*^AB^(**r**) for anisole–H_2_O (a) and for anisole–NH_3_ (b) complexes. The presented isosurfaces encompass 50%, 40%, 30%, 20%, 10%, and 1% of the integrated dispersion energy density difference Δ*Q*^AB^(**r**). Values on the color scale are reported in m*E*_h_·Å^–3^. Positive values correspond to regions where the dispersion energy density in the excited state is depleted (less negative) compared to the ground state. Basis set is aug-cc-pVTZ.

As discussed, both complexes experience a slight reduction in the dispersion attraction following vertical excitation. Visualizing these changes in real space using a recently introduced local dispersion density function^34^*Q*^*AB*^(**r**), where ∫*Q*^*AB*^(**r**) d**r** = *E*_disp_^(2)^, provides insight into the distribution of the energetic effect onto groups of atoms on both molecules. In Figure 2, we present differences between dispersion energy densities corresponding to excited and ground states (for comparison, see Figure S2 in the Supporting Information showing changes in the electron density induced by excitation). As one can infer from Figure 2, in the anisole–water complex, dispersion depletion involves the methoxy group of anisole and the lone-pair on oxygen. The effect is stronger in magnitude for the H···π contact in the second dimer, where π orbitals on the benzene ring interact with an evenly distributed dispersion density on ammonia.

Recall that the difference in interaction energies between excited and ground state dimers, Δ*E*_int_, is directly linked to shifts in excitation energies of the monomer on which the exciton is localized. Namely, if ω^(A^*^B)^ and ω^(A^*^)^ denote excitation energy of monomer A in the presence and absence, respectively, of monomer B, then the shift ω^(A^*^B)^ – ω^(A^*^)^ corresponds to Δ*E*_int_. In a high resolution laser-induced fluorescence experiment, Becucci et al.^47^ observed a blue shift of +0.54 m*E*_h_ (119 cm^–1^) upon formation of the anisole–water 1:1 complex. The corresponding change of the interaction energy predicted by SAPT(CAS) and CC methods are equal to +0.62 m*E*_h_ and +0.71 m*E*_h_, respectively. The ZPE correction for the water complex on the same set of geometries from (TD-)B3LYP calculations is small and amounts to ca. –0.04 m*E*_h_ (−0.1 kJ/mol, see Table 6 in ref (60)), which taken together leads to a good agreement of both SAPT and CC results with respect to the experimentally determined shift. Experimental study of the anisole–ammonia complex by Biczysko and coauthors^51^ revealed a red-shift of the anisole band equal to −0.91 m*E*_h_ (−199 cm^–1^). Recently, Loman et al. obtained a similar value of −0.88 m*E*_h_ (−193 cm^–1^).^53^ Without accounting for ZPE, the CC method predicts a red shift of −1.22 m*E*_h_. The agreement of SAPT(CAS) is only qualitative: the sign of the shift matches experimental observation, but the value, – 0.11 m*E*_h_, is underestimated. In contrast to the water complex, ZPE gives a sizable contribution to the shift for the ammonia complex. The ZPE value predicted at the MP2 level of theory and matching our choice of geometries is equal to −0.27 m*E*_h_ (see Table 5 in ref (51)). Thus, correcting for ZPE would bring the SAPT(CAS) shift closer to the experimental value, while worsening the agreement of the CC-based shift.

## 4 Conclusions

We have presented an in-depth SAPT study of intermolecular interactions in the ground S_0_ and electronically excited S_1_ states of anisole–water and anisole–ammonia complexes. The analysis has been performed with the SAPT(CAS) method, which is currently the only SAPT variant suitable for dimers with localized excitons. Motivated by previous experimental and theoretical works, we have focused on different behavior of these systems upon the S_1_ ← S_0_ transition: while the anisole–water binding becomes weaker following excitation, an opposite trend, i.e., stronger interaction, is observed for ammonia.

The ground state anisole–water dimer has a typical signature of a hydrogen-bond:^77^ although the electrostatic energy is the dominant attractive force, it is canceled almost entirely by the first-order exchange repulsion. As a result, both dispersion and induction contribute to the binding. In the excited state, the hydrogen bond is weakened—this results predominantly from a decrease in the electrostatic attraction. Geometry relaxation in the S_1_ state minimizes Pauli repulsion, but not enough to compensate for the reduced attraction. The SAPT(CAS) picture for the anisole–ammonia complex is subtler. In contrast to water, in the relaxed S_1_ state all energy components become larger in magnitude, with attractive forces overwhelming the increased repulsion. In this case, two major players are the first-order exchange and second-order dispersion. The analysis of the vertical S_1_ → S_0_ anisole–ammonia de-excitation shows that the stronger binding of the excited and relaxed S_1_ state, as compared to the S_0_ state at the same geometry, is predominantly due to reduced Pauli exchange.

The accuracy of the SAPT(CAS) interaction energies was verified against the CC results and experimental high-resolution spectroscopy data. SAPT(CAS) correctly predicts shifts of the S_0_ ← S_1_ anisole band induced by microsolvation. For the water complex, SAPT(CAS) shift closely matches both the experiment and the CC reference. In the case of ammonia, the agreement is only qualitative, i.e., SAPT(CAS) predicts stronger binding of the S_1_ state, but the magnitude of stabilization is underestimated. We attribute this to an insufficient treatment of intramonomer correlation by the chosen monomer wave functions.

## Data Availability

The raw data are available in the Zenodo repository at 10.5281/zenodo.12799514.

## Supporting Information Available

Anisole CAS(12,12) orbitals, differences of electron densities between the first vertically excited state and the ground state for anisole–water and anisole–ammonia complexes, total CASSCF energies of the studied systems, SAPT(CAS)/aug-cc-pVTZ results with CAS(6,6) active space on anisole and Hartree–Fock reference for the partner, SAPT(CAS)/aug-cc-pVTZ results obtained with the CAS(12,12) reference for anisole and CAS(8,8) wave functions for partners (PDF)
