# Supplementary material for: Anisole–Water and Anisole–Ammonia Complexes in Ground and Excited (S1) States: A Multiconfigurational Symmetry-Adapted Perturbation Theory (SAPT) Study
Source: J Phys Chem A. 2024 Oct 1;128(40):8816–24. doi: 10.1021/acs.jpca.4c04928 (PMC11480881; doi:10.1021/acs.jpca.4c04928)
Supplement: Supplementary file 1 — jp4c04928_si_001.pdf [file jp4c04928_si_001.pdf]

Supporting Information for
"Anisole-Water and Anisole-Ammonia Complexes in Ground and Excited (S_1) States: A Multiconfigurational SAPT Study"

Agnieszka Krzemińska,[1] Malgorzata Biczysko,[2] Katarzyna Pernal,[1,*] and Michał Hapka[3,†]

[1] *Institute of Physics, Lodz University of Technology, ul. Wolczanska 217/221, 93-005 Lodz, Poland*

[2] *Faculty of Chemistry, University of Wroclaw, F. Joliot-Curie 14, 50-383 Wroclaw, Poland*

[3] *Faculty of Chemistry, University of Warsaw, ul. L. Pasteura 1, 02-093 Warsaw, Poland*

[*] pernalk@gmail.com

[†] michal.hapka@uw.edu.pl

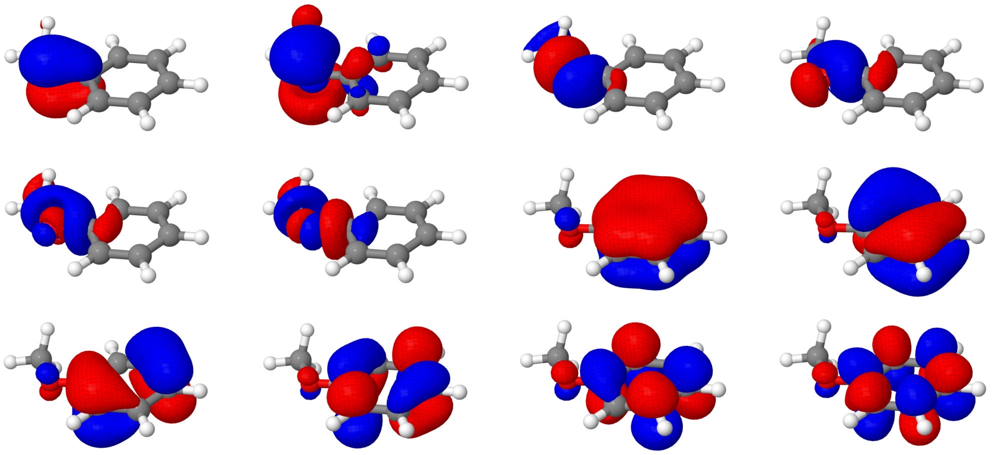

FIG. S1. Anisole CAS(12,12) orbitals: π(OC), π^*(OC), 2 σ_{CO}, 2 σ^*_{CO}, 3 π, 3 π^*. Basis set is aug-cc-pVTZ.

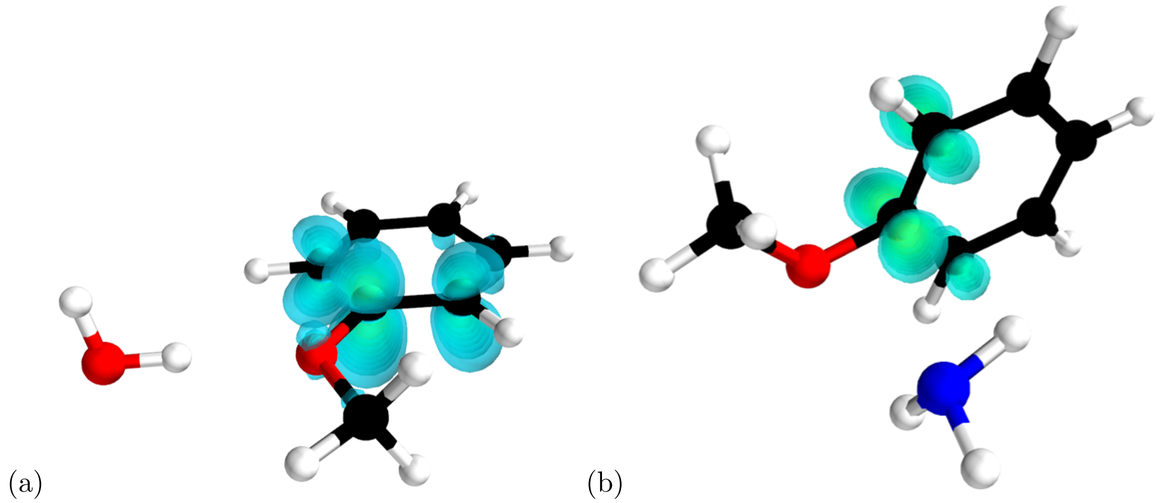

FIG. S2. Differences of electron densities corresponding to first vertical excited and ground states respectively, for anisole-H_2O (a) and anisole-NH_3 (b) complexes. Each isosurface consists of 6 contours of the difference density, generated to encompass 50%, 40%, 30%, 20%, 10% and 1% of the overall density, respectively.

TABLE S1. Total CASSCF energies (in Hartree) computed for anisole-H_2O and anisole-NH_3 complexes, where monomers are described with CAS(12,12) [anisole] or CAS(8,8) [water and ammonia] wave functions in their ground and excited states. Basis set is aug-cc-pVQZ.

	anisole	monomer
	anisole - H_2O	
ground-state	−344.890 572	−76.196 744
ground-state in adiabatic geom.	−344.882 603	−76.196 748
excited-state vertical	−344.709 148	−76.196 744
excited-state adiabatic	−344.713 867	−76.196 748
	anisole - NH_3	
ground-state	−344.889 485	−56.334 222
ground-state in adiabatic geom.	−344.880 000	−56.334 254
excited-state vertical	−344.710 324	−56.334 222
excited-state adiabatic	−344.711 783	−56.334 254

TABLE S2. Anisole-H_2O: SAPT(CAS) interaction energy decomposition with the CAS(6,6) active space on anisole and Hartree-Fock reference for water. In the notation X/Y (1st column), X denotes either the ground (GS) or the excited (ES) state, Y refers to either the ground (GS) or the relaxed excited state (AD) geometry. Δ(vert.) are differences between ES/GS and GS/GS results. Δ(adiab.) are differences between ES/AD and GS/GS results. Energy unit is milliHartree. Basis set is aug-cc-pVTZ.

	$E^{(1)}_{\rm elst}$	$E^{(1)}_{\rm exch}$	$E^{(2)}_{\rm ind}$	$E^{(2)}_{\rm exch-ind}$	$\delta_{\rm HF/CAS}$	$E^{(2)}_{\rm disp}$	$E^{(2)}_{\rm exch-disp}$	$E_{\rm int}$	$E_{\rm int}+\delta$
GS/GS	−12.206	11.021	−4.892	2.675	−1.445	−5.641	0.894	−8.149	−9.594
ES/GS	−12.087	10.977	−4.865	2.663	−1.435	−5.633	0.891	−8.053	−9.488
GS/AD	−9.852	7.922	−3.323	1.728	−0.884	−4.980	0.687	−7.818	−8.702
ES/AD	−9.875	7.903	−3.326	1.739	−0.879	−4.974	0.686	−7.848	−8.727
Δ (vert.)	0.119	−0.044	0.027	−0.011	0.010	0.008	−0.003	0.096	0.115
Δ (adiab.)	2.331	−3.118	1.566	−0.936	0.565	0.667	−0.208	0.302	0.867

TABLE S3. Anisole-NH_3: SAPT(CAS) interaction energy decomposition with CAS(6,6) active space on anisole and Hartree-Fock reference for ammonia. In the X/Y notation (1st column), X denotes either the ground (GS) or the excited (ES) state, Y refers to either the ground (GS) or the relaxed excited state (AD) geometry. Δ(vert.) are differences between ES/GS and GS/GS results. Δ(adiab.) are differences between ES/AD and GS/GS results. Energy unit is milliHartree. Basis set is aug-cc-pVTZ.

	$E^{(1)}_{\mathrm{elst}}$	$E^{(1)}_{\mathrm{exch}}$	$E^{(2)}_{\mathrm{ind}}$	$E^{(2)}_{\mathrm{exch-ind}}$	$\delta_{\mathrm{HF/CAS}}$	$E^{(2)}_{\mathrm{disp}}$	$E^{(2)}_{\mathrm{exch-disp}}$	E_{int}	$E_{\mathrm{int}} + \delta$
GS/GS	−5.371	5.498	−1.892	1.289	−0.439	−5.951	0.733	−5.694	−6.133
ES/GS	−5.110	5.326	−1.840	1.251	−0.435	−5.868	0.711	−5.530	−5.965
GS/AD	−7.475	10.355	−4.187	3.064	−1.020	−7.640	1.300	−4.583	−5.603
ES/AD	−7.333	9.780	−3.887	2.771	−0.959	−7.464	1.223	−4.909	−5.868
Δ (vert.)	0.261	−0.173	0.052	−0.037	0.004	0.084	−0.023	0.165	0.169
Δ (vert.) without dispersion:									0.107
Δ (adiab.)	−1.962	4.282	−1.995	1.483	−0.520	−1.512	0.490	0.785	0.265
Δ (adiab.) without dispersion:									1.288

TABLE S4. Anisole-H_2O: SAPT(CAS) interaction energy decomposition with the CAS(12,12) active space on anisole and CAS(8,8) for water. In the X/Y notation (1st column), X denotes either the ground (GS) or the excited (ES) state, Y refers to either the ground (GS) or relaxed excited state (AD) geometry. Δ(vert.) are differences between ES/GS and GS/GS results. Δ(adiab.) are differences between ES/AD and GS/GS results. Energy unit is milliHartree. Basis set is aug-cc-pVTZ.

	$E^{(1)}_{\rm elst}$	$E^{(1)}_{\rm exch}(S^2)$	$E^{(2)}_{\rm ind}$	$E^{(2)}_{\rm exch-ind}$	$\delta_{\rm HF/CAS}$	$E^{(2)}_{\rm disp}$	$E^{(2)}_{\rm exch-disp}$	$E_{\rm int}$	$E_{\rm int}+\delta$
GS/GS	−11.487	11.549	−5.024	2.959	−1.445	−5.829	0.913	−6.919	−8.364
ES/GS	−11.077	11.467	−4.964	2.950	−1.427	−5.814	0.905	−6.533	−7.960
GS/AD	−9.307	8.225	−3.378	1.885	−0.884	−5.126	0.699	−7.002	−7.886
ES/AD	−9.180	8.165	−3.350	1.886	−0.887	−5.110	0.692	−6.897	−7.784
Δ (vert.)	0.410	−0.082	0.060	−0.009	0.018	0.015	−0.008	0.386	0.404
Δ (adiab)	2.307	−3.384	1.674	−1.073	0.558	0.719	−0.221	0.022	0.580

TABLE S5. Anisole-NH_3: SAPT(CAS) interaction energy decomposition with CAS(12,12) active space on anisole and CAS(8,8) for ammonia. In the X/Y notation (1st column), X denotes either the ground (GS) or the excited (ES) state, Y refers to either the ground (GS) or the relaxed excited state (AD) geometry. Δ(vert.) are differences between ES/GS and GS/GS results. Δ(adiab.) are differences between ES/AD and GS/GS results. Energy unit is milliHartree. Basis set is aug-cc-pVTZ.

	$E^{(1)}_{\rm elst}$	$E^{(1)}_{\rm exch}(S^2)$	$E^{(2)}_{\rm ind}$	$E^{(2)}_{\rm exch-ind}$	$\delta_{\rm HF/CAS}$	$E^{(2)}_{\rm disp}$	$E^{(2)}_{\rm exch-disp}$	$E_{\rm int}$	$E_{\rm int}+\delta$
GS/GS	−5.548	6.018	−2.140	1.537	−0.439	−6.243	0.797	−5.579	−6.018
ES/GS	−5.376	5.909	−2.120	1.529	−0.435	−6.186	0.782	−5.462	−5.897
GS/AD	−8.106	11.418	−4.989	3.872	−1.020	−8.101	1.441	−4.465	−5.485
ES/AD	−8.141	10.828	−4.690	3.559	−0.959	−7.938	1.362	−5.017	−5.976
Δ (vert.)	0.172	−0.109	0.020	−0.008	0.004	0.057	−0.015	0.117	0.121
Δ (adiab.)	−2.593	4.810	−2.550	2.022	−0.520	−1.695	0.565	0.562	0.042
